# Measuring the mechanical properties of molecular conformers

**DOI:** 10.1038/ncomms9338

**Published:** 2015-09-21

**Authors:** S. P. Jarvis, S. Taylor, J. D. Baran, N. R. Champness, J. A. Larsson, P. Moriarty

**Affiliations:** 1 School of Physics & Astronomy, University of Nottingham, Nottingham NG7 2RD, UK; 2 Department of Chemistry, University of Bath, Claverton Down, Bath BA2 7AY, UK; 3 School of Chemistry, University of Nottingham, Nottingham NG7 2RD, UK; 4 Applied Physics, Division of Materials Science, Department of Engineering Sciences and Mathematics, Luleå University of Technology, SE-971 87 Luleå, Sweden

## Abstract

Scanning probe-actuated single molecule manipulation has proven to be an exceptionally powerful tool for the systematic atomic-scale interrogation of molecular adsorbates. To date, however, the extent to which molecular conformation affects the force required to push or pull a single molecule has not been explored. Here we probe the mechanochemical response of two tetra(4-bromophenyl)porphyrin conformers using non-contact atomic force microscopy where we find a large difference between the lateral forces required for manipulation. Remarkably, despite sharing very similar adsorption characteristics, variations in the potential energy surface are capable of prohibiting probe-induced positioning of one conformer, while simultaneously permitting manipulation of the alternative conformational form. Our results are interpreted in the context of dispersion-corrected density functional theory calculations which reveal significant differences in the diffusion barriers for each conformer. These results demonstrate that conformational variation significantly modifies the mechanical response of even simple porpyhrins, potentially affecting many other flexible molecules.

## Abstract (web summary)

Manipulation of single molecules can be achieved using scanning probe microscopy but the influence of molecular conformation on this process has, until now, been unclear. Here, the authors probe two different types of porphyrin conformer on a surface and see strong differences in their mechanochemical response.

## Graphical abstract

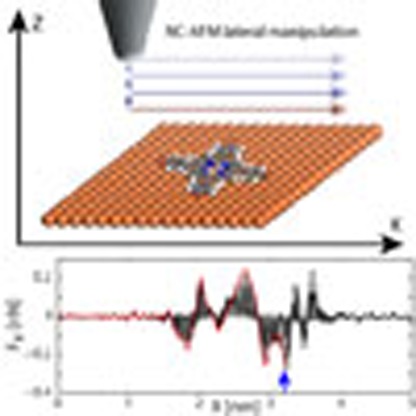

When two molecules of the same chemical composition differ in structure via rotations around intramolecular bonds—that is, the breaking and reforming of bonds is not required to superimpose the two structures—we describe the molecules as conformers^1^. This has very important consequences as the conformation of a molecule can affect key physicochemical properties such as conductance, reactivity, and optical absorption/emission and can be especially important in biological systems^2^,^3^. Scanning probe microscopy enables conformation to be switched and examined on an individual molecule basis, providing fascinating insights into mechanochemical changes. With scanning tunnelling microscopy (STM) one can identify static molecular conformers^4^ and examine their physiochemical properties^5^,^6^,^7^. In addition, a wide range of STM experiments demonstrating molecular manipulation have now been reported including rolling^8^,^9^,^10^, translating^11^ and switching of molecules^12^,^13^,^14^. Scanning probe studies of molecules capable of adopting multiple conformations, however, have been much fewer in number^15^,^16^,^17^,^18^, and there has not been an attempt to date to elucidate the differences in the response of different conformers to tip-induced forces.

To investigate the chemomechanical properties of a conformer during manipulation, it is essential that the interaction between the scanning probe and the molecule is purely mechanical. Non-contact atomic force microscopy (NC-AFM), with its sensitivity to force rather than current, is ideally suited to this task. Although atomic manipulation with NC-AFM is now well established^19^, manipulation of molecules is still very much an emerging field of study. Whilst a small number of groups have reported molecular manipulation with NC-AFM (for example^20^,^21^), very few have recorded the forces involved, and generally only relatively simple molecules have been investigated.

The lateral force responsible for moving metal adatoms and CO molecules was first quantified by Ternes *et al.*^22^. Since then, larger molecules, such as perylene-tetracarboxylic-dianhydride (PTCDA)^23^,^24^ and H_2_Pc (ref. 25) have been investigated. These studies, however, examine either small or flat molecules which are structurally rigid and so have significantly limited internal degrees of freedom compared with other commonly investigated large molecules. Consequently, it remains an open question as to whether the force for manipulation can be measured for complex molecular structures, and, in particular, whether conformational dependencies can be identified.

In this article, we report the forces required to laterally manipulate two conformers of a single molecule on Cu(111). The prototypical molecule we study is tetra(4-bromophenyl) porphyrin (Br_4_TPP) which, similar to a number of phenyl-terminated porphyrin molecules, is known to adopt two different conformations on noble metal (111) surfaces^26^. We show that the lateral forces required for manipulation have a strong dependence on conformation type, even though each conformer shares almost identical adsorption characteristics. Nudged elastic band (NEB) calculations using van der Waals density functional theory (vdW-DFT) reveal that despite a similar adsorption energy for each conformer, the energy barriers for diffusion increase by over 50% for one conformational form.

## Results

### Conformer structure imaged with non-contact atomic force microscopy

**Figure 1:**
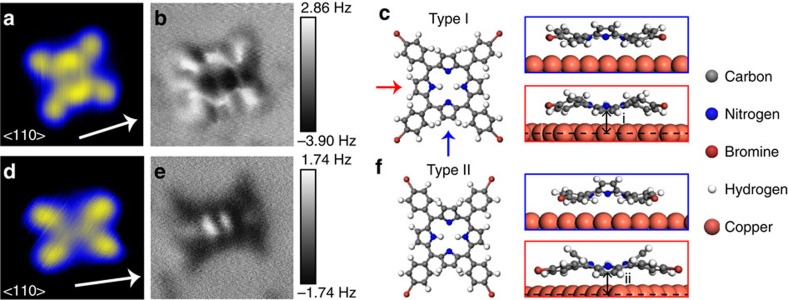
STM and NC-AFM images of Br_4_TPP conformers on Cu(111). Type I conformers are shown imaged in the (**a**) constant current STM and (**b**) constant height NC-AFM modes. Equivalent scans for the Type II conformer are shown in **d**,**e**. DFT calculated structures, shown in **c**,**f** reveal significant differences in distortion for the Type I and II conformers, respectively. Ball-and-stick illustrations are shown from a top-down view (surface removed for clarity) and two side-on views marked with red and blue arrows and boxes. The C–Cu adsorption distance, marked as i and ii is 2.70 and 2.74 Å for the Type I and II conformers, respectively. STM data were collected in separate experiments. Parameters: STM: (A)-1V/4pA, (**d**)+1V/50pA. AFM: *a*_0_=200 pm, V=50 mV. Image sizes 3.5 × 3.5 nm. STM images are shown with a saturated colour scale.

In Fig. 1 we show typical constant current STM and constant height NC-AFM imaging of conformers deposited on the Cu(111) surface at 77 K, and imaged at 5 K (see Methods for details). As discussed in detail elsewhere^27^ the Br_4_TPP molecule (shown in Fig. 1) adopts two different conformations on Cu(111) via the alignment and interaction of the porphyrin macrocycle and phenyl leg groups with the underlying copper. The greatest contribution to the van der Waals (vdW) interaction comes from the flexible phenyl ‘leg' groups at each corner of the molecule. This gives rise to strong distortions within the molecular core of both conformers, which adopt a saddle arrangement to provide better positioning of the legs with respect to the underlying copper substrate. The two Br_4_TPP conformers, which we denote Type I and Type II, are shown as ball-and-stick diagrams in Fig. 1c,f, respectively. The key feature to note is that while both conformers adopt a similar saddle structure, in which the two iminic pyrrole groups become tilted towards the underlying copper surface, in the Type II arrangement the distortion is more extreme. The reduced steric repulsion between the pyrrole and phenyl groups therefore allows the phenyl legs to ‘swing' into more favourable locations to maximize the vdW interaction.

The observed conformers of Br_4_TPP are similar in appearance to those observed by Iancu *et al.*^28^ for the structurally similar TBrPP-Co, which, although initially thought to differ as saddle and planar structures, were later found to both adopt a saddle arrangement^29^,^30^ similar to that found for the Br_4_TPP molecule we study here.

NC-AFM, which is sensitive to the tip-sample force gradient, can directly image features relating to the intramolecular structure of a molecule^31^,^32^,^33^, as well as the structure of molecular conformers^34^. This can be achieved via termination of the scanning probe with a single molecule^35^ or spontaneous termination of the tip^36^. Figure 1b,e show NC-AFM images taken in a similar manner for Type I and II conformers, respectively. We expect that in this case our tip is molecularly terminated, either by another Br_4_TPP molecule or potentially with CO, due to residual gas contaminants in the vacuum chamber (Supplementary Figs 1 and 3, and related discussion).

The primary feature observed for the Type I molecule in Fig. 1b is the strong repulsion located over the phenyl leg groups and what is assumed to be two of the pyrrole units at the top and bottom of the molecule. Despite the evidence for molecular tip termination, intramolecular resolution within the aromatic rings is not observed. We note that other non-planar molecules, such as sexi-phenyl (ref. 37), appear with similar resolution. The consistent magnitude of the Δ*f* signal observed across the molecule corresponds well to the calculated structure in Fig. 1c, which shows similar heights of the distorted core and leg groups. In contrast, the Type II conformer shown in Fig. 1e exhibits a significantly more repulsive core than the periphery of the molecule. This is typically observed as two bright features corresponding to the expected location of the tilted pyrrole groups within the macrocycle (see Supplementary Fig. 2 for other examples) and is likely a direct consequence of the buckled conformational structure (Fig. 1f).

### Lateral force measurements

**Figure 2:**
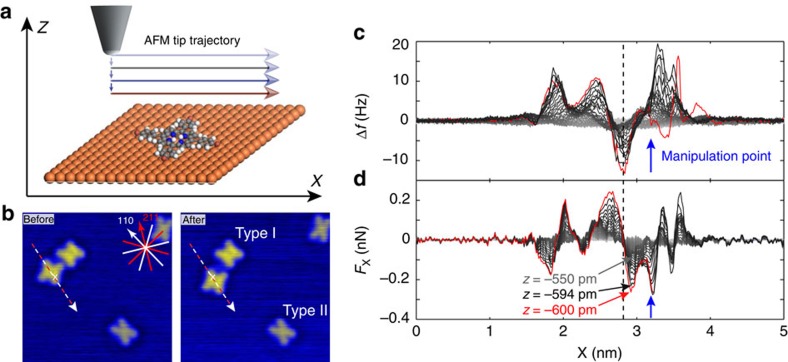
Lateral manipulation of the Type I Br_4_TPP conformer. (**a**) Schematic demonstrating the manipulation protocol used to build a two-dimensional (2D) force-map. (**b**) STM images taken before and after manipulation of a single Type I molecule along a direction 15° off from the <211> crystal direction. (**c**) Overlaid line profile plot of the short-range 2D Δf during manipulation. Darker line profile traces correspond to smaller tip-sample separations. Molecular manipulation occurs during the final trace at *z*=−600 pm (red line), marked by the blue arrow. The tip-sample separation (*z*) is defined relative to the initial Δ*f* trace at 0 pm (STM setpoint positioned on the molecule was at *z*=−400 pm) (**d**) Plot of the lateral forces obtained from the data shown in **c**.

The lateral force required to move a molecule is directly related to its adsorption on the surface and the associated potential energy landscape. We therefore measured the lateral force required to displace each conformer during multiple manipulation events. By ensuring that the tip remained unchanged throughout the experiment, the forces for each conformer can be directly compared. We followed a similar protocol to Ternes *et al.*^22^ where a two-dimensional map of Δ*f* was measured as the tip was incrementally approached and scanned across the molecule in a fixed direction. After each trace, the tip was returned to its original location at an increased height to ensure that manipulation could only occur when the tip is moving in the desired direction (Fig. 2a). Following collection of the two-dimensional map of Δ*f*(*x*, *z*) the long-range background was subtracted using an ‘off' spectrum and a conversion made to *U*(*x*, *z*) (ref. 38) allowing the lateral, *F*_*x*_, and vertical, *F*_*z*_, forces to be calculated.

In Fig. 2b a typical manipulation experiment is shown where a Type I conformer is laterally translated with the NC-AFM tip by two lattice spacings. As described below, successful manipulation was generally only observed for metallic tips providing poorer STM resolution. To rapidly image large areas between manipulation events the molecules were scanned in constant current STM mode with the oscillating tip. During the manipulation, however, the bias voltage was reduced to 0 V, minimizing the tunnelling current. We found that the Type I conformer could be translated along the <110> and <211> directions or in the directions 15° off between the two (see Supplementary Fig. 4 and Supplementary Movie 1 for full manipulation sequence). In all but two manipulation attempts the molecule is translated in the same direction as the NC-AFM tip trajectory. We note that tip trajectories matching the direction of the molecular diagonal (as shown in Fig. 2b) were significantly more efficient at translating the molecule than other directions, which would often cause undesired rotations.

The recorded Δ*f* map is shown in Fig. 2c as an overlaid line profile plot. At the point of manipulation we observe a jump in the Δ*f* trace corresponding to translation of the molecule, marked by the blue arrow (white cross in Fig. 2b). To quantify the forces responsible for the manipulation we plot the corresponding *F*_*x*_ profile, as shown in Fig. 2d. By measuring the value of *F*_*x*_ at the position marked by the blue arrow we observe that the manipulation occurred at the point of maximum attractive lateral force measured as −0.26(5) nN. The uncertainty in *F*_*x*_ is estimated from the uncertainty in the oscillation amplitude, which is assumed to dominate other potential sources of error such as the spring constant, and errors arising from phase lock loop (PLL) control (see SI). Out of the seven attempts at manipulation of the free molecule (Supplementary Fig. 4F–L) that resulted in translational motion we obtained a range of *F*_*x*_ varying from −0.2 to −0.34 nN, independent of the surface crystal direction, whereas the measured values of *F*_*z*_ varied from −0.25 to +0.7 nN. Consequently, it seems highly likely that lateral forces dominate the manipulation we report.

### Identification of manipulation mechanism

**Figure 3:**
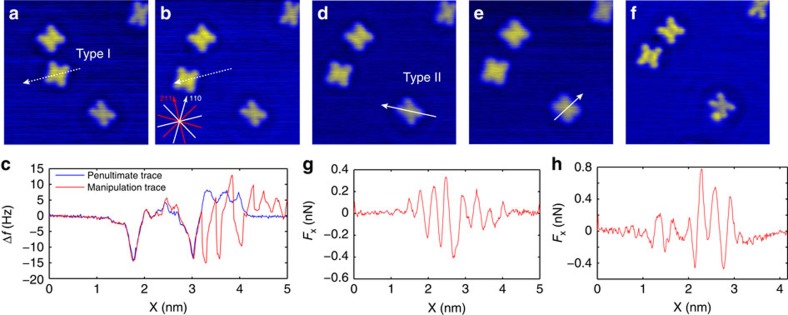
Evidence for ‘pull' manipulation and manipulation attempts on the Type II Br_4_TPP conformer. STM images recorded (**a**) before and (**b**) after long-range manipulation spanning several lattice sites. (**c**) Δ*f* line traces recorded immediately before and during manipulation. (**d**–**f**) Manipulation attempts for a Type II conformer, taken during the same experimental session. The extracted lateral forces for (**d**,**e**) are shown in **g** and for (**e**,**f**) shown in **h**. In each case the AFM tip is modified before the Type II Br_4_TPP conformer could be manipulated. This is visible as a subtle degradation in image quality from (**d**–**e**), and by the deposition of tip material from (**e**–**f**).

Further insight into the manipulation mechanism can be gained by examining extended manipulation events across several lattice sites. Figure 3a,b shows one such manipulation event, taken from the same experimental session, where the Type I conformer is translated seven lattice spacings along the <110> surface direction. In Fig. 3c characteristic jumps are observed during the final Δ*f* trace. These are separated by ∼2.6 Å, corresponding to the Cu(111) atomic spacing that determines the adsorption geometry of both Br_4_TPP conformers. Moreover, the manipulation trace exhibits the distinctive ‘sawtooth' pattern often observed in STM experiments of adsorbate manipulation^39^. It is important to note that the frequency shift decreases in magnitude when the tip is positioned over an adsorbate (in contrast to the tunnel current). As such our data are interpreted as a distinctive ‘pull' manipulation, in agreement with our extracted attractive lateral forces. We observe, however, that not all manipulation traces show this behaviour, similar to reports on other molecules^24^.

The assignment of a pulling mechanism is in agreement with previous work by Ternes *et al.*, who measured much smaller forces of 17 and 160 pN for Co and CO, respectively, also on Cu(111) (ref. 22). Interestingly Langewisch *et al.*^23^report a pushing mechanism for PTCDA on Ag(111). However, their method differed in that the tip was only laterally approached up to the centre point of the molecule, potentially preventing the tip being able to pull the molecule at larger tip-sample separations.

### Conformational dependence of lateral force

To measure the difference in lateral force required to manipulate the alternative Type II conformer we continued experiments using the same tip termination, once again across various crystal directions as shown in Fig. 3d–f. In this case, surprisingly, we were unable to instigate lateral manipulation without also incurring changes in the tip structure (Fig. 3d–f.). As a result, the lateral forces could only be extracted up until the point where a tip change was observed, thus providing a minimum value for *F*_*x*_. Repeated measurements in a single experimental session produced values for *F*_*x*_ ranging from −0.3 to −0.7 nN. In each experiment the tip was modified before the Type II conformer could be moved, even to the extent that tip material was deposited on the surface as in Fig. 3f. Example lateral force plots for D–E and E–F are shown in G and H, respectively. Based on the range of measured *F*_*x*_ we estimate that the threshold force for manipulation is 1.5–3 times greater for the Type II conformer as compared with the Type I. We note that, similar to previous reports^28^, molecules are sometimes observed exhibiting a mixed conformation, with half of the molecule in the Type I arrangement and the other appearing as Type II. Although attempts were made to manipulate such mixed conformers, they were found to be extremely unstable and always switched to an entirely Type II arrangement before manipulation could be achieved.

It should be noted that variations in tip structure between experiments can have a significant effect on measurements of *F*_*x*_, in some cases resulting in values as low as 0.1 nN for the Type I conformer. It has previously been shown that tip-induced barrier collapse is a key mechanism in atomic manipulation with NC-AFM^40^,^41^. As a result we expect that the NC-AFM tip also strongly affects the barriers involved in molecular manipulation. Therefore it is only by taking multiple measurements for both molecular conformers with the same tip structure that we are able to directly compare the threshold forces for manipulation.

Repeated measurements for a range of unknown tip structures led to significant variation in tip stability. Successful manipulation was most commonly achieved for sharp tips, characterized by 
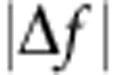
 values 
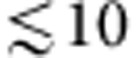
 Hz during dynamic STM measurements, resulting in a success rate of ∼80% and ∼12% for the Type I and II conformers, respectively. This produced the range of forces described above. In addition, the most stable tip states—that is, those capable of repeated manipulations—generally produced lower resolution STM images (for example, the increased spatial resolution in Fig. 3f). Finally, many of the tip structures were unable to laterally manipulate the molecule. Instead, and despite following an identical manipulation protocol, molecules were often rotated, and in some rare cases switched between conformations. We propose that this behaviour can be explained by either metallic or molecularly terminated tips. Whereas it is known that metallic tips can attractively ‘pull' an adsorbed molecule, molecularly passivated tips are expected to have a significantly suppressed interaction, restricting them to ‘push' type manipulations. Due to the large size of the molecule and the relatively high flexibility of the phenyl leg groups, we expect ‘push' type manipulations are most likely to destabilize and rotate the molecule.

### Calculated barriers for diffusion

**Figure 4:**
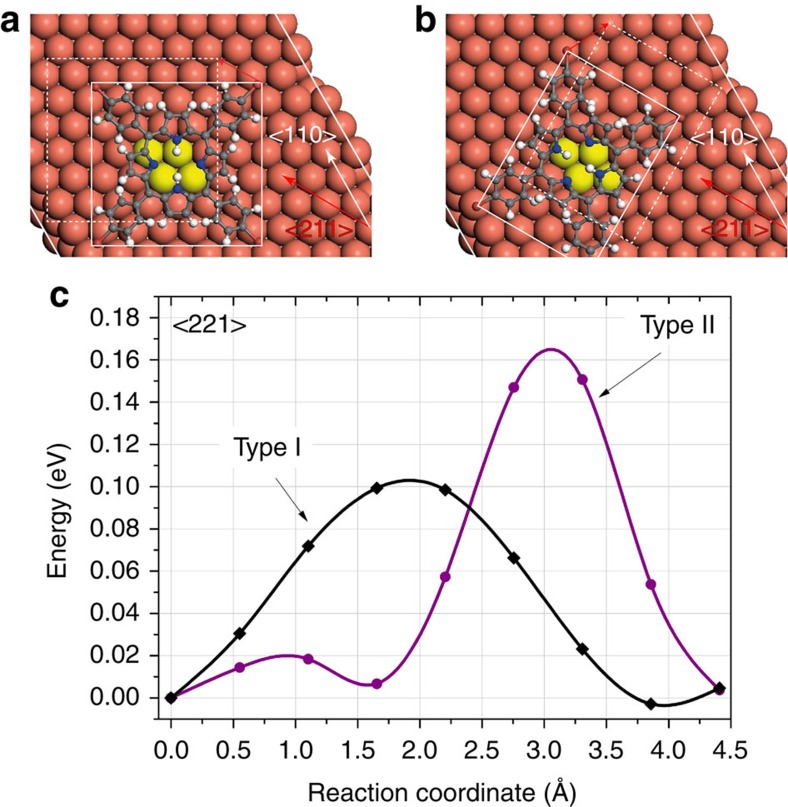
NEB calculated energy barriers for translation. Calculated barriers for (**a**) the Type I and (**b**) Type II conformers along the <211> surface direction. Ball-and-stick schematics (**a**,**b**) highlight the manipulation direction with the solid(dashed) box illustrating the initial (final) position of the molecule. Calculated energy barriers (**c**) reveal a significantly higher energetic cost for translation of the Type II conformer. The reaction coordinate corresponds to the total distance that the molecule travels between the staring and final NEB point.

In our DFT calculations a subtle interplay between the preference of the phenyl legs to sit closer to the copper and the increased strain of the molecular core ultimately leads to very similar values for the binding energy of each molecule, calculated as −4.1 and −3.9 eV for the Type I and II conformers, respectively. To understand the manipulation we therefore calculated the energy barriers for molecular diffusion using vdW-DFT and the NEB method. To facilitate comparison with experiment we modelled translations of each conformer in both the <110> and <211> directions between equivalent adsorption positions on the underlying Cu(111) surface. Ball-and-stick schematics, shown with the corresponding energy barriers for translation along the <211> direction, are presented in Fig. 4a,b for the Type I and II conformer, respectively. From our calculations we determine barriers of ∼0.105 eV and ∼0.170 eV for the Type I and II conformers, respectively, corresponding to an approximate increase by a factor of ∼1.6. We note that the energy barriers were found to be functional independent, with multiple dispersion-corrected functionals, including the DFT-D3 and optB86b-vdW methods all showing similar increases in the calculated energy barrier. This is a clear indication that the Type II conformer has a higher threshold for movement relative to the Type I arrangement, following the trend observed in our experiments.

The primary contribution to the vdW adsorption energy originates from the cumulative interaction between the atoms of the molecule with the surface copper. This is shown in Fig 4 for the starting stable structures, where the phenyl and pyrrole rings predominantly occupy positions above copper bridge sites, maximizing the carbon atoms alignment with the surface copper atoms, thus maximizing the vdW interaction. The variation between conformers in the calculated barrier is therefore likely due to the increased distortion of the Type II conformer, which serves to reduce the degree of overlap between stable adsorption sites. We note that in our calculations the molecules show very little flexibility during translation. The importance of dispersion interactions has been highlighted in a number of recent reports^42^,^43^, where in some cases the adsorption was found to depend almost entirely on vdW interactions^44^.

Due to the unknown nature of the NC-AFM tip a tip cluster was not included in our calculations, which instead models the diffusion of the free molecule on the Cu(111) surface. We are therefore unable to account for any potential difference in the interaction the tip might have from one conformer to another, which could play an additional role in modifying the energy barriers for manipulation. We also note that the small size of the calculated barriers imply that the molecules should diffuse at relatively low temperatures, despite experimental evidence confirming that the molecules are stable at 77 K (refs 26, 27). In addition to the known difficulties^45^,^46^,^47^ in determining adsorption energies using vdW-DFT methods, this is potentially due to the absence of zero point energy corrections in our calculations, which has been observed to significantly increase the size of calculated diffusion barriers^48^ for single pyrrole molecules (a major constituent of Br_4_TPP). Unfortunately, however, due to the large size of the Br_4_TPP molecule, zero point energy calculations are extremely costly and could not be performed. As a consequence, making quantitative comparisons between experiment and simulation is challenging, and we instead compare the overall trends, which provide important insight into the manipulation process.

As mentioned above, our measurements for the Type I conformer show no clear variation in the lateral forces required for translation along the <110> and <211> directions. This observation is supported by additional NEB calculations along the <110> direction which show almost identical barriers for diffusion along the <211> direction shown above (Supplementary Fig. 5). We suggest that the similarity in the potential energy surface (PES) along each crystal direction is potentially due to the large vdW interaction between the Br_4_TPP molecule and the Cu(111) surface which dominates the adsorption energy. In contrast, in single atom adsorbate motion, site specific chemical interactions are expected to have more directional dependence along the so-called ‘hard' and ‘easy' surface directions. Therefore, in the case of the Br_4_TPP molecule, the highest point of the barrier simply corresponds to the point of minimum alignment between the carbon and copper atoms, that is, where the vdW interaction is weakest.

In summary, we directly measure the conformational dependence of a large, flexible, organic molecule during lateral manipulation. Lateral force measurements show that molecular conformation is capable of driving much larger changes in the mechanical response than expected. This will likely have significant implications for other much larger organic molecules that exhibit variations in conformational structure, both adsorbed on surfaces and in solution.

## Method

### Scanning probe microscopy

Measurements were taken on a Createc GmbH LT STM-AFM system operating under ultrahigh vacuum conditions (base pressure <6 × 10^−11^ mbar) cooled to 5 K. Clean Cu(111) surfaces were prepared as described elsewhere^27^. Br_4_TPP molecules were deposited on the Cu(111) sample (cooled to 77 K) using a homemade crucible resistively heated to ∼350±50 °C. A commercial qPlus sensor (Createc GmbH) with a separate tunnel current wire was used for both the STM and NC-AFM experiments (*f*_0_∼20 kHz; Q∼30,000 at 5 K; nominal spring constant 1,800 Nm^−1^). Tungsten tips were electrochemically etched and were prepared via controlled tip crashing and bias voltage pulsing until good STM/NC-AFM resolution was achieved. As a result, the tips are expected to be copper-, rather than tungsten, terminated. (We also discuss the possibility of molecularly terminated tips in the main text).

### Electronic structure theory modelling

DFT calculations were performed using the non-local correlation functional optB86b-vdW (ref. 49) (vdW-DFT) that approximately accounts for dispersion interactions as implemented within the projector-augmented wave method^50^ in the VASP software package^51^. An energy cutoff of 400 eV and a Gaussian smearing of 0.1 eV were used for the initial electronic occupations. Energy barriers were calculated using the climbing image NEB method^52^,^53^.

## Additional information

**How to cite this article:** Jarvis, S. P. *et al.* Measuring the mechanical properties of molecular conformers. *Nat. Commun.* 6:8338 doi: 10.1038/ncomms9338 (2015).

## Supplementary Material

Supplementary Information Supplementary Figures 1-8, Supplementary Methods and Supplementary References

Supplementary Movie 1 Movie of full manipulation sequence for a Type I Br4TPP conformer.
