# Supplementary material for: Measuring the mechanical properties of molecular conformers
Source: Nat Commun. 2015 Sep 21;6:8338. doi: 10.1038/ncomms9338 (PMC4595718; doi:10.1038/ncomms9338)
Supplement: Supplementary Information — Supplementary Figures 1-8, Supplementary Methods and Supplementary References [file ncomms9338-s1.pdf]

SUPPLEMENTARY FIGURES

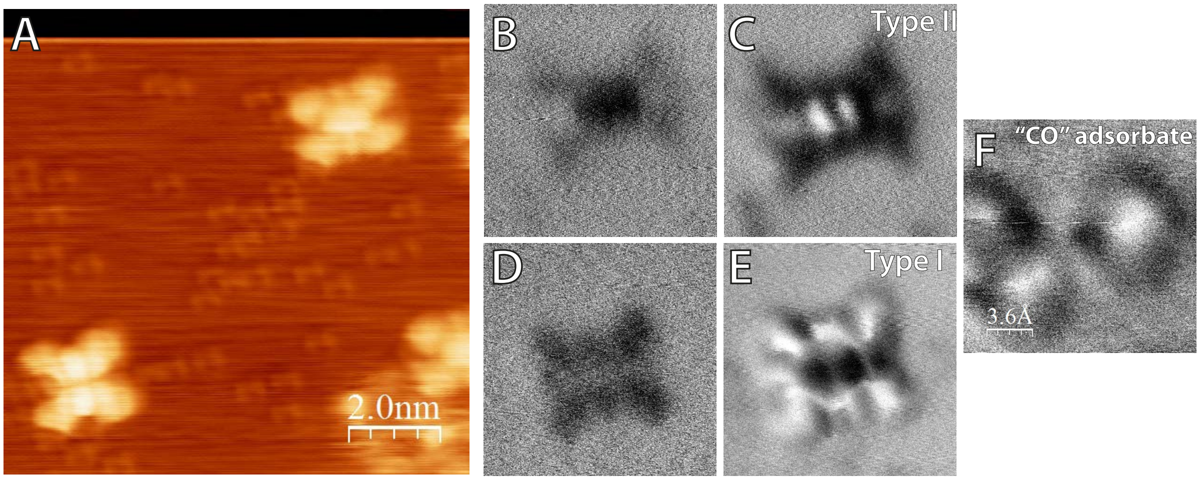

Supplementary Figure 1. STM and additional NC-AFM data for Br_4TPP data in Figure 1. (A) Constant current STM image of the two molecular conformers. A large number of potentially adsorbed CO molecules are also present. (B) Constant height frequency shift image for the Type II conformer at the initial onset of the attractive interaction and (C) at lower tip-sample separations during the onset of repulsive contributions (Z=-80pm relative to B). (D) Constant height frequency shift image for the Type I conformer at the same tip-sample separation as B. (E) Image taken at smaller tip-sample separation (Z=-150pm relative to B and D). (F) Constant height images of local "CO" adsorbates show relatively symmetric tip structure supporting the assignment of a sharp passivated tip (Z=-420pm relative to B). Parameters: *STM: (A)-1V/20pA. AFM:* a_0*=200pm, V=50mV. (B-E)Image sizes 3.5×3.5nm.*

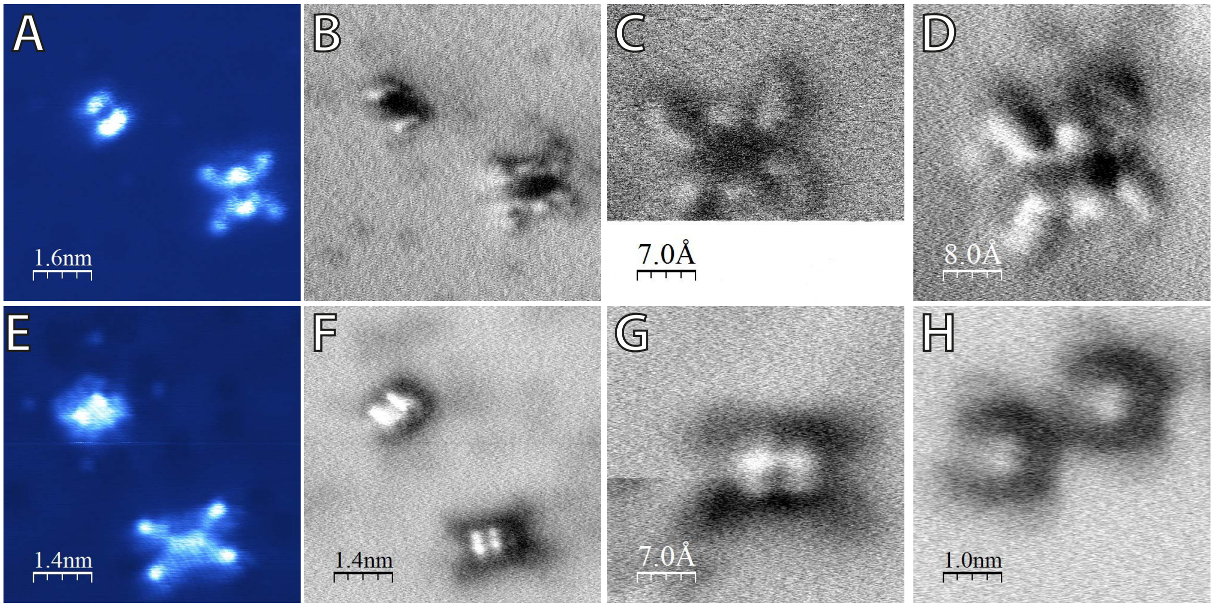

Supplementary Figure 2. Additional NC-AFM images of Br_4TPP conformers. (A) Constant height current image of an intact and fully de-brominated Type I conformer. (B) Corresponding frequency shift image. (C,D) Additional frequency shift images across different experiments showing similar structure. (E) Constant height current image for intact and fully de-brominated Type II conformer. (F) Corresponding frequency shift image. (D,H) Additional frequency shift images showing similar core structure.

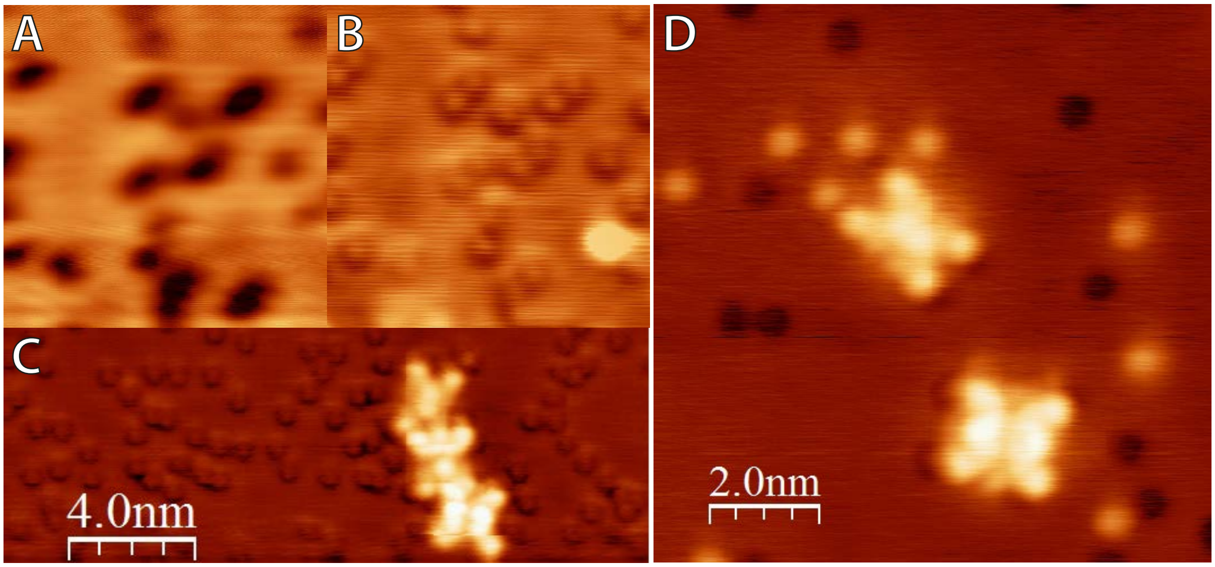

Supplementary Figure 3. Inversion of molecular adsorbate contrast in STM. (A) Before and (B) after images of surface adsorbates taken during successive STM images. (C) Larger STM image taken directly after B. (D) STM showing Br_4TPP conformers, Cu adatoms and the distinctive adsorbate features.

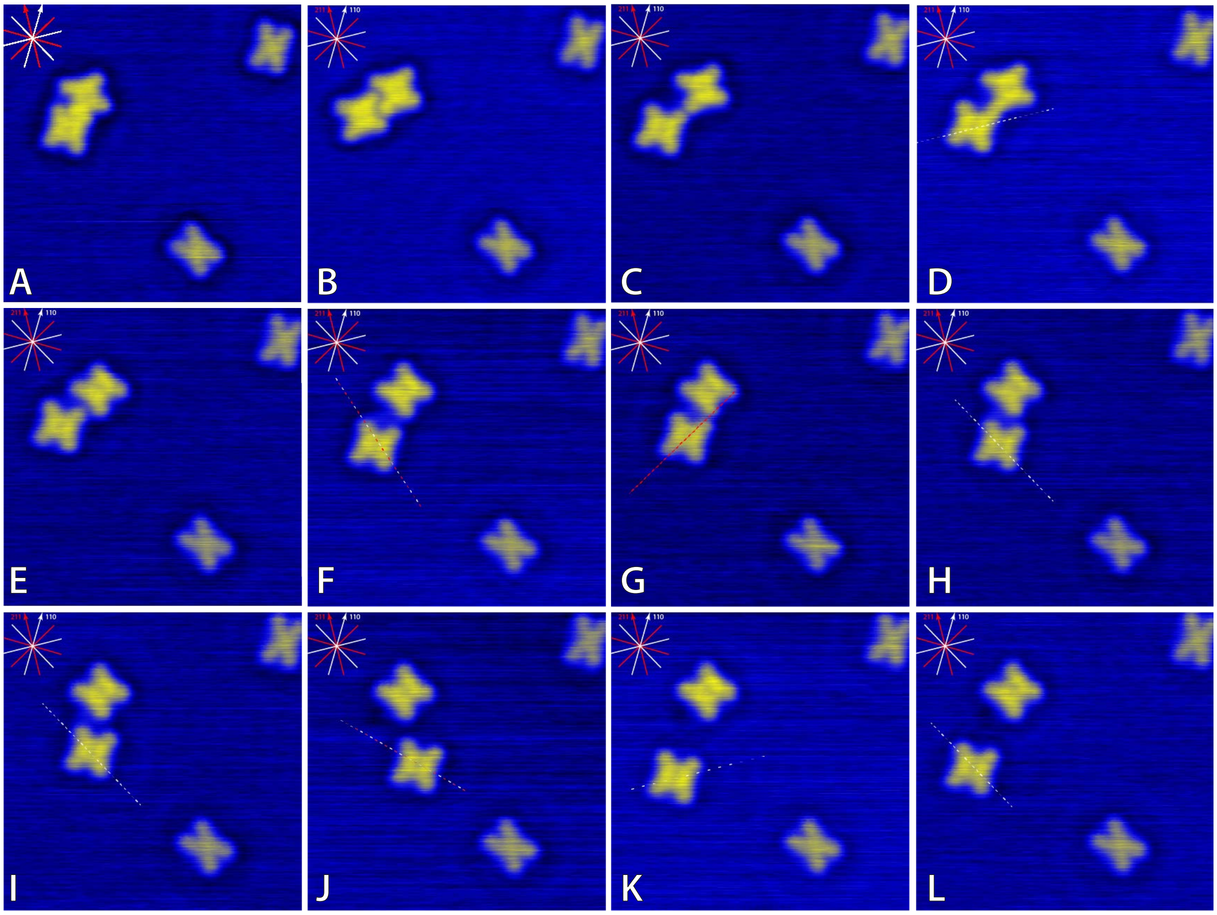

Supplementary Figure 4. Complete sequence of Type I conformer manipulations. After freeing the molecule in panels (A-E) the molecule was laterally translated seen times (F-L). White and red dashed lines indicate translation in <110> and <211> directions respectively, mixed red and white lines indicate translations 15° between the two crystal directions.

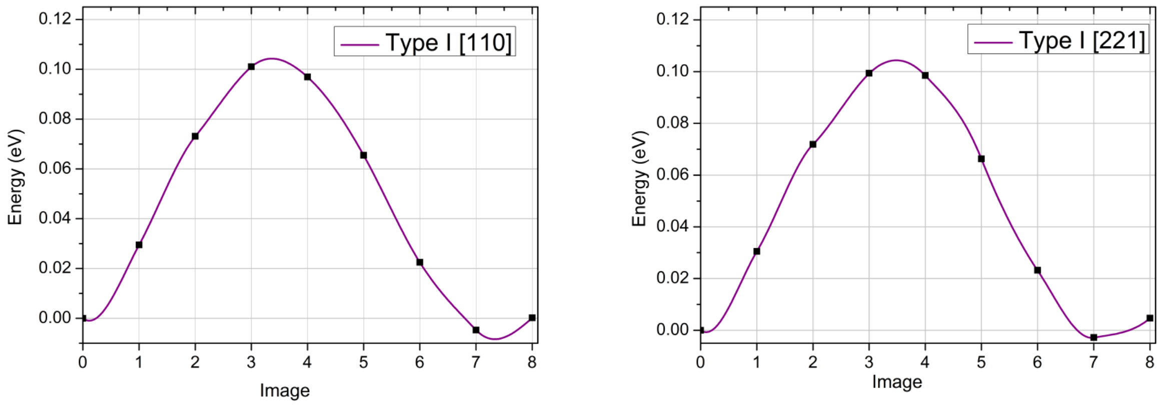

Supplementary Figure 5. Nudged elastic band energy barrier calculations for translation of the Type I conformer in the <110> and <211> surface directions.

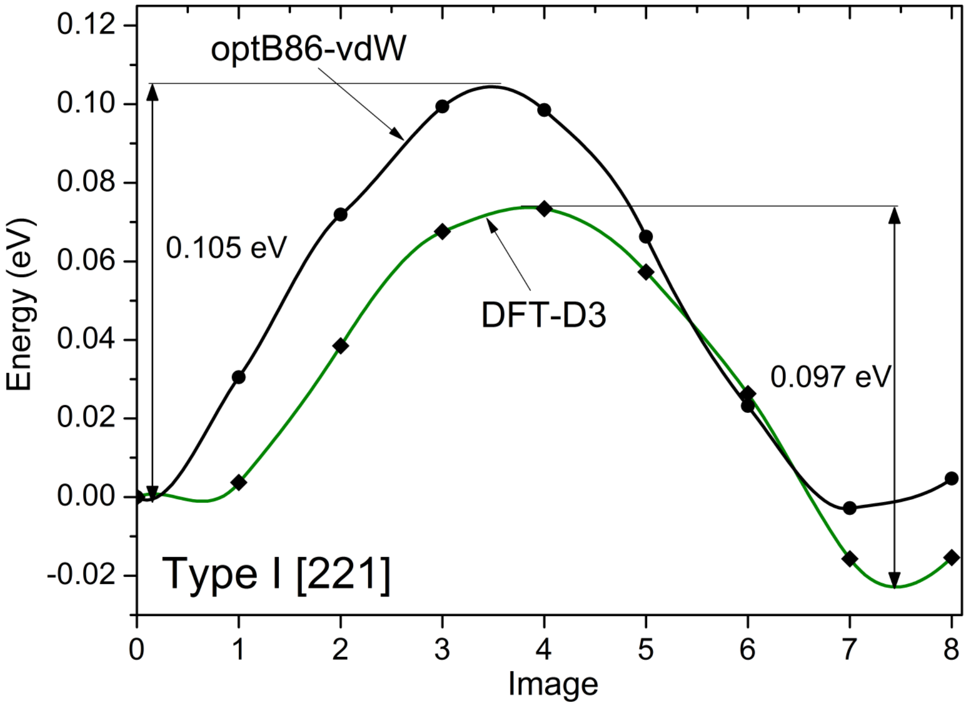

Supplementary Figure 6. Nudged elastic band energy barrier calculations comparing the optB86-vdW and DFT-D3 functionals for translation of the Type I conformer in the <211> direction.

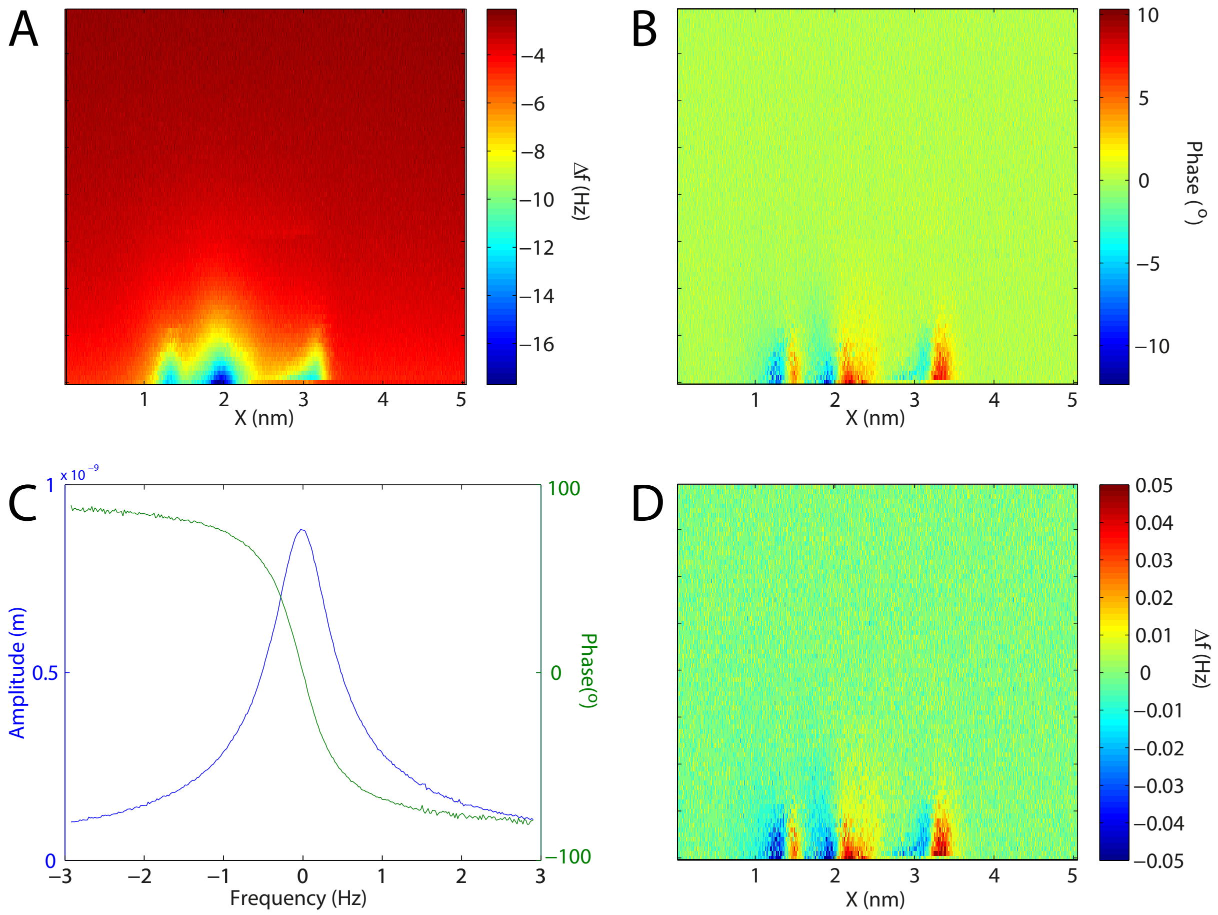

Supplementary Figure 7. Phase error analysis during lateral manipulation. (A) Example Δf data during manipulation. (B) Associated phase error. (C) Frequency sweep taken for the tuning fork at free resonance. (D) Approximated frequency error due to phase error calculated from C.

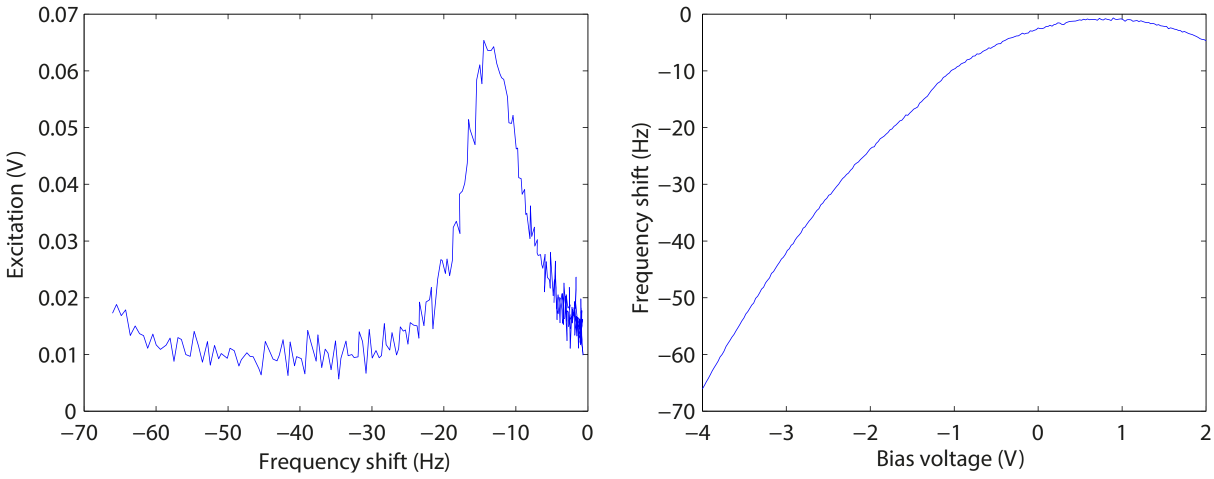

Supplementary Figure 8. Transfer function analysis. Plot of tuning fork excitation vs frequency shift during $\Delta f(V)$ measurement 30nm retracted from the surface.

SUPPLEMENTARY METHODS

Additional simulation methods

To model the molecule-surface interface, a 12 × 12 hexagonal three-layer-thick slab of Cu(111) was used and the Br_4TPP molecule was placed parallel to the surface using a thick vacuum level of 20.0 Å to ensure that there was no spurious interaction between periodically repeated slabs in the normal direction to the surface. Lateral movement of the Br_4TPP molecule on the surface was performed using climbing image nudge elastic band (NEB) method [1, 2]. During NEB calculations the first two layers of the surface slab model and the molecule are fully relaxed until the force acting on each atom is less than 0.02 eV/Å , whereas the bottom slab layer was kept fixed in the bulk position. The Brillouin zone sampling was performed at the Γ-point in all calculations. We have also tested (2,2,1) k-point mesh for the calculations, but the results showed negligible difference from those using the Γ-point. Dependence of calculated energy barriers on the chosen functional was also tested by performing NEB calculations with the DFT-D3[3] vdW correction on top of the generalized gradient approximation (GGA) parametrization by Perdew Burke Ernzerhof (PBE), for the Type I conformer translated in the <211>-direction. In this case no significant difference in the energy barrier was found between these two different approaches to describe dispersion, see Figure S6.

Phase errors during 2D Δf measurement

Maintaining optimal PLL feedback is essential during collection of the lateral $\Delta f(x, z)$ data. For normal measurements of $\Delta f(z)$, variations in Δf are gradual, and generally follow a smooth Lennard-Jones like profile which can easily be tracked by the PLL. The tip trajectory when taking $\Delta f(x)$ measurements, however, is significantly different. In this case rapid variations in Δf can occur due to varying reactivity across a surface or molecule. Therefore as the tip laterally moves across the surface the PLL must track sudden changes in Δf which can lead to large errors in phase regulation, even with significantly increased bandwidth. To reduce the effect of phase errors on the recorded Δf channel we increased the phase bandwidth from 15Hz (image acquisition) to 40Hz until deviations in the phase were reduced to ∼10° or below. In Figure S7 we attempt to quantify the effect of the phase error(B) on the recorded Δf(A) measured during a manipulation attempt. In principle the

relationship between phase and frequency shift can be readily determined from a frequency sweep measurement as shown in Figure S7 C. If we approximate the Q of the tuning fork to remain unchanged as the tip interacts with the surface, an estimate for the phase-induced frequency error can be made as shown in Figure S7 D. Due to the high Q factor for the tuning fork at 5K temperatures and the high PLL bandwidths the error is shown to be minimal ($\lesssim$0.05Hz). It is important to note, however, that at higher temperatures this error will be much more pronounced. For instance, frequency sweeps measured at 77K temperatures (which reduce the Q to $\sim$8000) show this error will increase to several 100mHz, thus becoming significant enough to affect calculated forces.

Apparent dissipation

In all measurements the oscillation amplitude was maintained at a constant value. Therefore in principle non-conservative dissipation can be measured. In the majority of cases where the oscillation excitation was recorded, dissipation above the molecule was not observed. Only in a minority of cases, particularly for the much larger frequency shifts recorded above Type II molecules, did we observe significant dissipation signals. Examination of the transfer function, however, demonstrated that this was largely due to apparent dissipation. Labuda *et al*[4] have convincingly demonstrated that the transfer function of the piezoacoustic excitation system is rarely flat, leading to measurements of apparent dissipation at specific frequency shift values unrelated to physical tip-sample processes. In Figure S8 we show an *in situ* measurement of the transfer function recorded for a qPlus sensor immediately following measurements that recorded dissipation. It is clear that a significant peak in apparent dissipation is recorded between Δf values of 10-20Hz. This corresponds exactly to the Δf recorded above the Br_4TPP molecule, suggesting that the recorded dissipation signal has no physical origin. It is therefore clear that apparent dissipation, in addition to the phase channel, must always be analysed to make meaningful conclusions from measured dissipation.

SUPPLEMENTARY REFERENCES

---

[1] Henkelman, G. & Jonsson, H. A climbing image nudged elastic band method for finding saddle points and minimum energy paths. *J. Chem. Phys.* **113**, 9901 (2000).

[2] Henkelman, G. & Jonsson, H. Improved tangent estimate in the nudged elastic band method for finding minimum energy paths and saddle points. *J. Chem. Phys.* **113**, 9978 (2000).

[3] Grimme, S., Chemie, T. O. & Münster, O.-c. I. D. U. Semiempirical GGA-Type Density Functional Constructed with a Long-Range Dispersion Correction. *J. Comput. Chem.* **16** (2006).

[4] Labuda, A., Miyahara, Y., Cockins, L. & Grütter, P. H. Decoupling conservative and dissipative forces in frequency modulation atomic force microscopy. *Physical Review B* **84**, 125433 (2011).
